# Supplementary material for: Assessing the Lifetime Cost-Effectiveness of Low-Protein Infant Formula as Early Obesity Prevention Strategy: The CHOP Randomized Trial
Source: Nutrients. 2019 Jul 19;11(7):1653. doi: 10.3390/nu11071653 (PMC6682975; doi:10.3390/nu11071653)
Supplement: Supplementary file 1 [file nutrients-11-01653-s001.pdf]

Supplemental Material

Assessing the lifetime cost-effectiveness of low-protein infant formula as early obesity prevention strategy: the CHOP randomized trial

Diana Sonntag, PhD[1,2], Freia De Bock, MD, MPH[1,3], Martina Trotzauer, MStat,[4], Berthold Koletzko, Prof, MD[4]

[1] Mannheim Institute of Public Health, Social and Preventive Medicine, Mannheim Medical Faculty of the Heidelberg University, Mannheim, Germany

[2] Department of Health Sciences, University of York, York, UK

[3] Federal Centre for Health Education, Cologne, Germany

[4] Department of Pediatrics, Dr. von Hauner Children's Hospital, University Hospital, LMU Munich, Germany

Figure S1: Flowchart of children from study entry to 6 years of age by study group

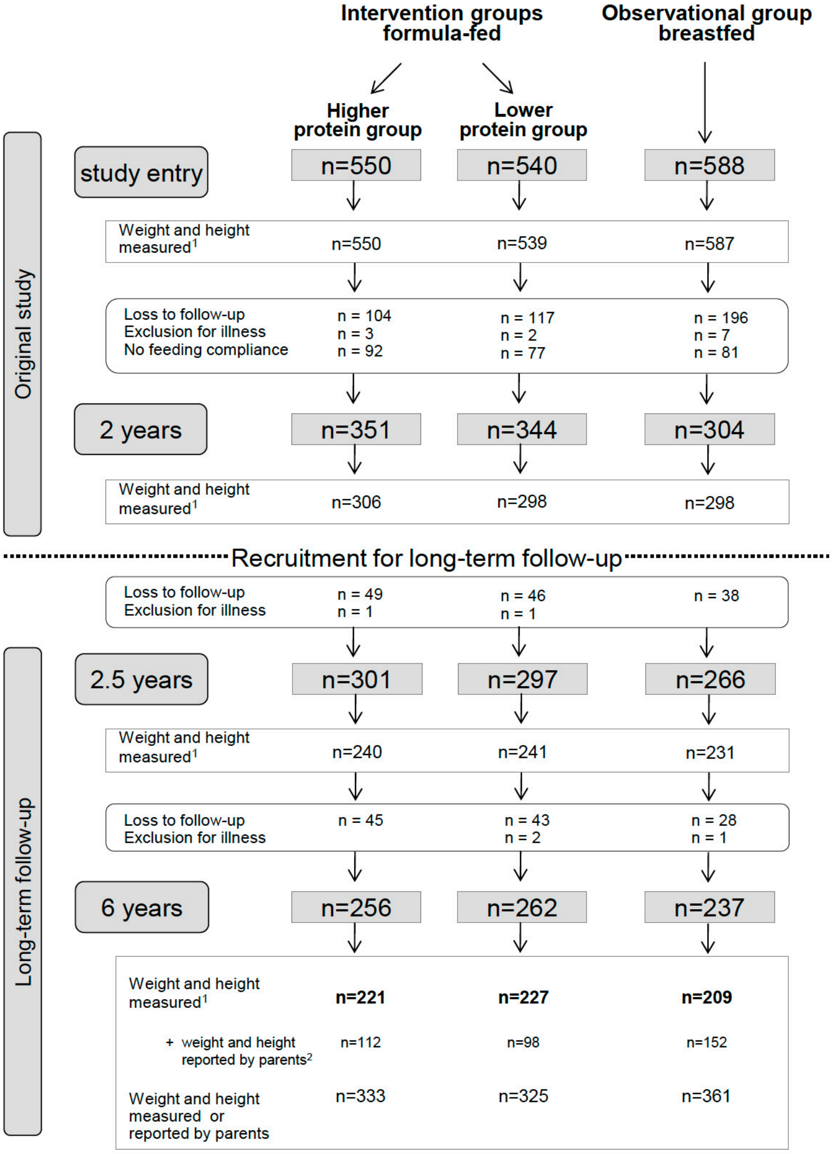

[1]height and weight measured at the study center by study personel, BMI calculated
[2]height and weight as reported by parents collected by telephone interview, BMI calculated

Table S1a: Epidemiological parameter values for Markov model

Model Parameter	Parameter Value		Range used for Univariate Sensitivity Analysis	Source
**I. Transition Probabilities**				
**Normal weight to Overweight**				
Cycle:	male	female		
1	0.02	0.01		
10	0.00	0.00		
20	0.03	0.01		
30	0.03	0.01		
40	0.02	0.02		
50	0.02	0.03		
60	0.00	0.01		
70	0.00	0.00		
80	0.00	0.00		
90	0.00	0.00		
**Overweight to Obese**				
Cycle:	male	female		
1	0.21	0.12		
10	0.03	0.04		
20	0.02	0.03		
30	0.01	0.02		
40	0.01	0.01		
50	0.01	0.02		
60	0.00	0.00		
70	0.00	0.00		
80	0.00	0.00		
90	0.00	0.00		
			(None)	We estimated transition probabilities by following Sonntag et al. (2015).
**Overweight to Normal weight**				
Cycle:	male	female		
1	0.00	0.00		
10	0.03	0.05		
20	0.00	0.00		
30	0.00	0.00		
40	0.00	0.00		
50	0.00	0.00		
60	0.00	0.00		
70	0.01	0.01		
80	0.00	0.00		
90	0.00	0.00		
**Obese to Overweight**				
Cycle:	male	female		
1	0.00	0.00		
10	0.00	0.00		
20	0.00	0.00		
30	0.00	0.00		
40	0.00	0.00		
50	0.00	0.00		
60	0.01	0.00		
70	0.02	0.02		
80	0.03	0.03		
90	0.05	0.04		
**II. Relative Risk**				
of mortality if obese in childhood				
male	1.10		(None)	Engeland et al. (2004)
female	1.30			
of mortality if overweight in adulthood	*agegroup 25-59:* 0.83			
	agegroup 60-69: 0.95			
	agegroup >70: 0.91		(None)	Flegal et al. (2005)
of mortality if obese in adulthood	*agegroup 25-59:* 1.36			
	agegroup 60-69: 1.26			
	agegroup >70: 1.07			

Table S1b: Cost data for Markov model

Model Parameter	Parameter Value			Range used for univariate Sensitivity Analysis	Source
**III. Costs per capita, sex and BMI**					
	BMI<25	25<BMI<30	BMI>30		
**Male:**					
15-20 years	0 €	2 €	54 €		
20-25 years	0 €	6 €	111 €		
25-30 years	0 €	7 €	85 €		
30-35 years	0 €	11 €	112 €		
35-40 years	0 €	17 €	140 €		
40-45 years	0 €	31 €	216 €		
45-50 years	0 €	52 €	307 €		
50-55 years	0 €	83 €	459 €		
55-60 years	0 €	62 €	344 €		
60-65 years	0 €	86 €	406 €		
65-70 years	0 €	113 €	488 €		
70-75 years	0 €	139 €	591 €		
75-80 years	0 €	165 €	672 €		
80-85 years	0 €	191 €	710 €		
85-90 years	0 €	192 €	649 €		
> 90 years	0 €	134 €	461 €		
				costs are increased by a factor of 2.1 at maximum	Konnopka et al. (2010)
**Female:**					
15-20 years	0 €	2 €	183 €		
20-25 years	0 €	8 €	284 €		
25-30 years	0 €	11 €	288 €		
30-35 years	0 €	17 €	311 €		
35-40 years	0 €	24 €	280 €		
40-45 years	0 €	42 €	281 €		
45-50 years	0 €	72 €	319 €		
50-55 years	0 €	114 €	426 €		
55-60 years	0 €	89 €	313 €		
60-65 years	0 €	126 €	334 €		
65-70 years	0 €	160 €	385 €		
70-75 years	0 €	215 €	456 €		
75-80 years	0 €	298 €	602 €		
80-85 years	0 €	346 €	655 €		
85-90 years	0 €	327 €	622 €		
> 90 years	0 €	218 €	440 €		

Figure S2a: Lifetime development of overweight and obese health states in the female population of Germany, stratified by infant feeding strategy

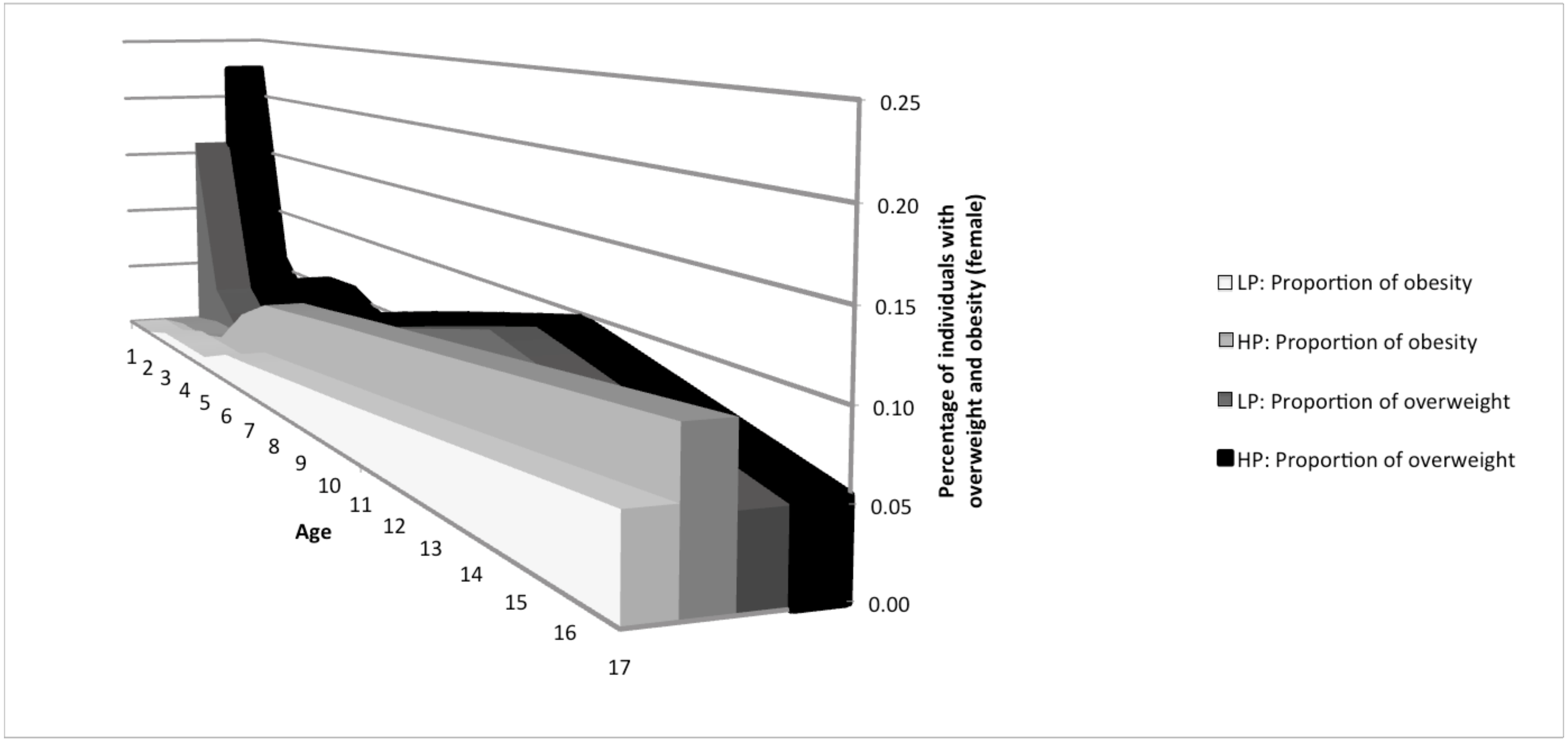

This figure presents the development of overweight and obesity health states for the female population in Germany for both infant feeding types.

Figure S2b: Lifetime development of overweight and obese health states in the male population of Germany, stratified by infant feeding strategy

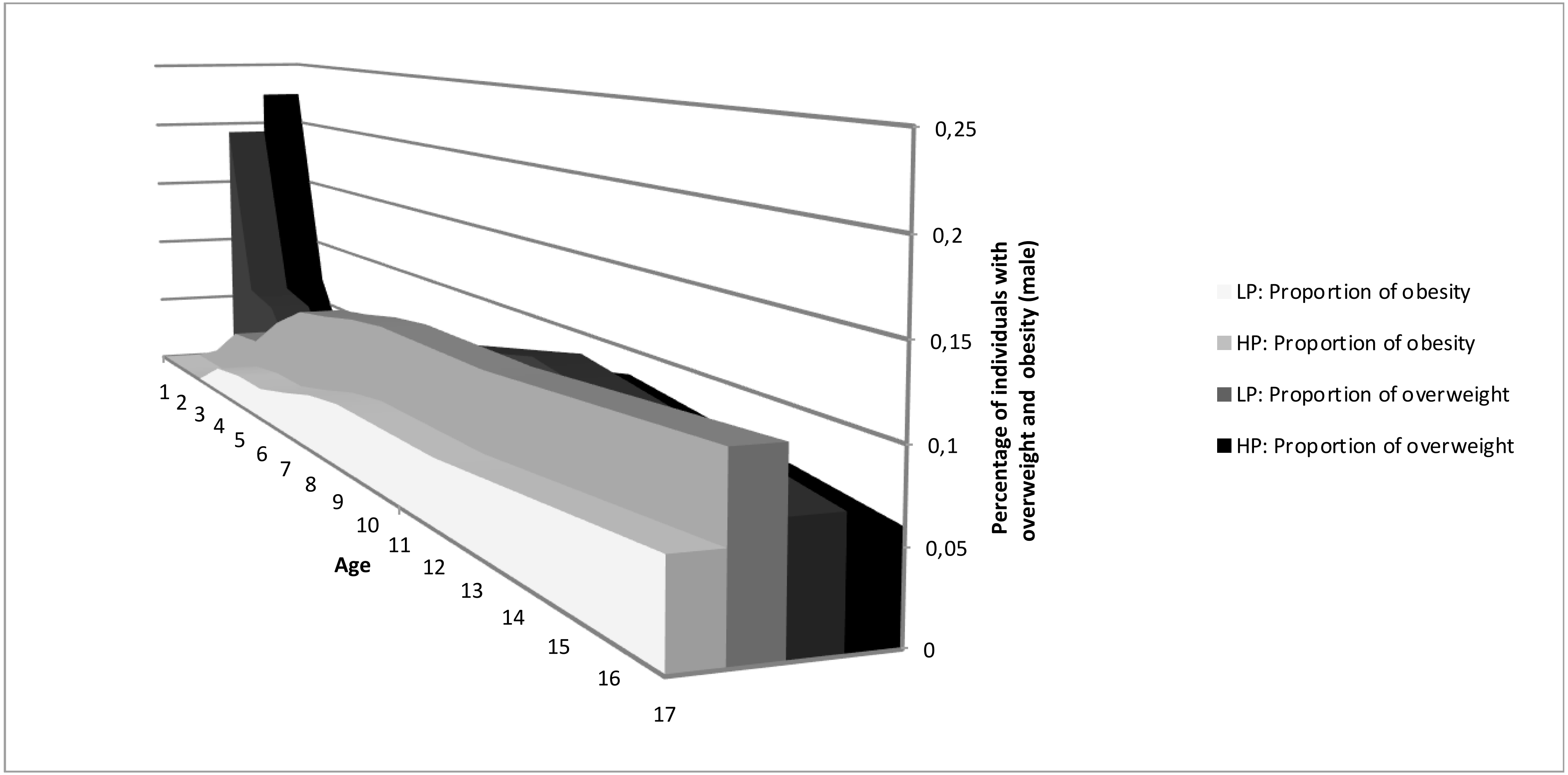

This figure presents the development of overweight and obesity health states for the male population in Germany for both infant feeding types.

Figure S3a: Cost-effectiveness acceptability curves of LP and HP content formula

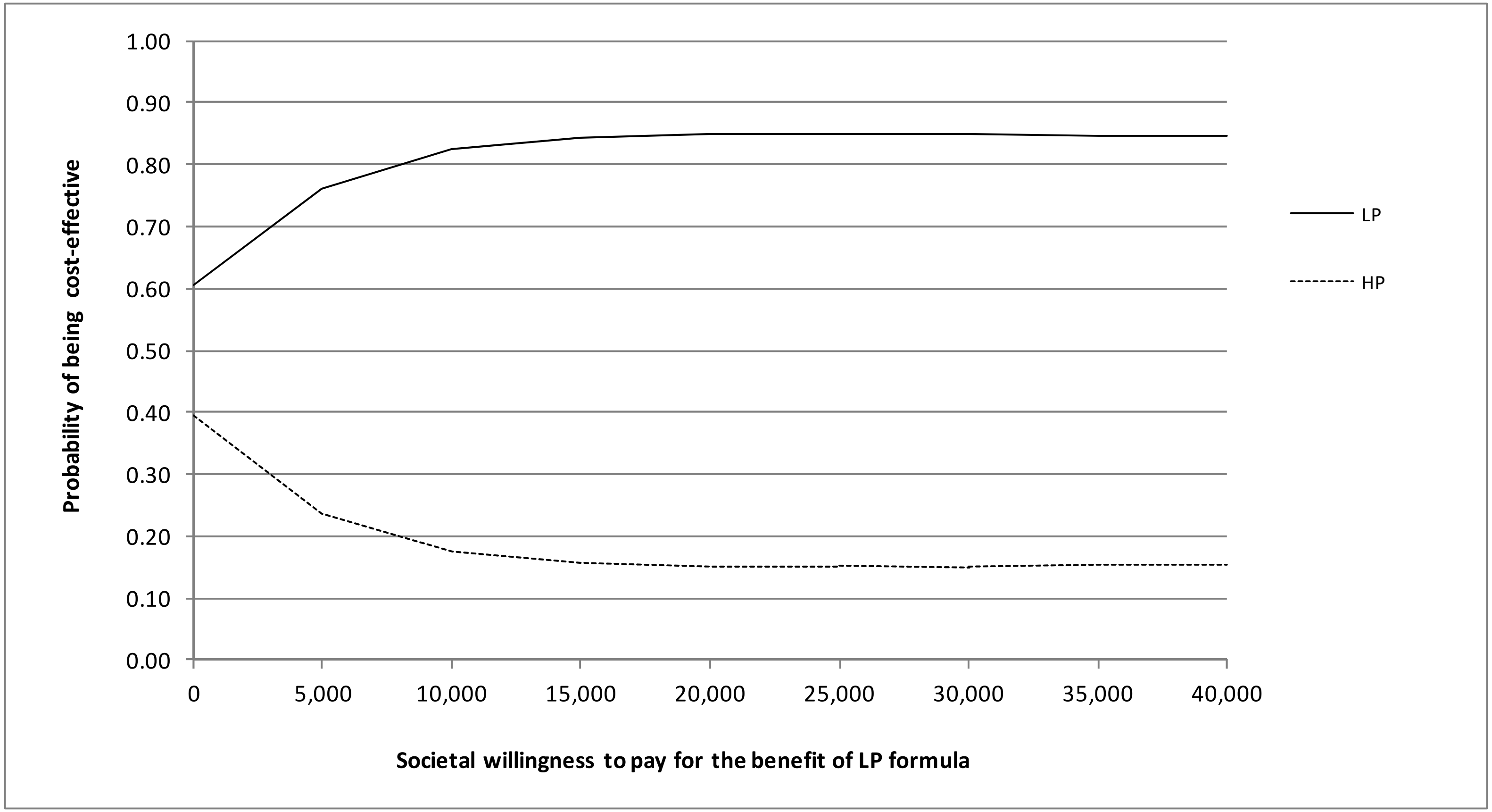

The cost-effectiveness acceptability curves (CEAC) show the probability that the LP formula is cost-effective compared with the HP formula for a range of maximum monetary values that the society might be willing to pay for a particular unit change in BMI.

Figure S3b: Results from univariate sensitivity analyses

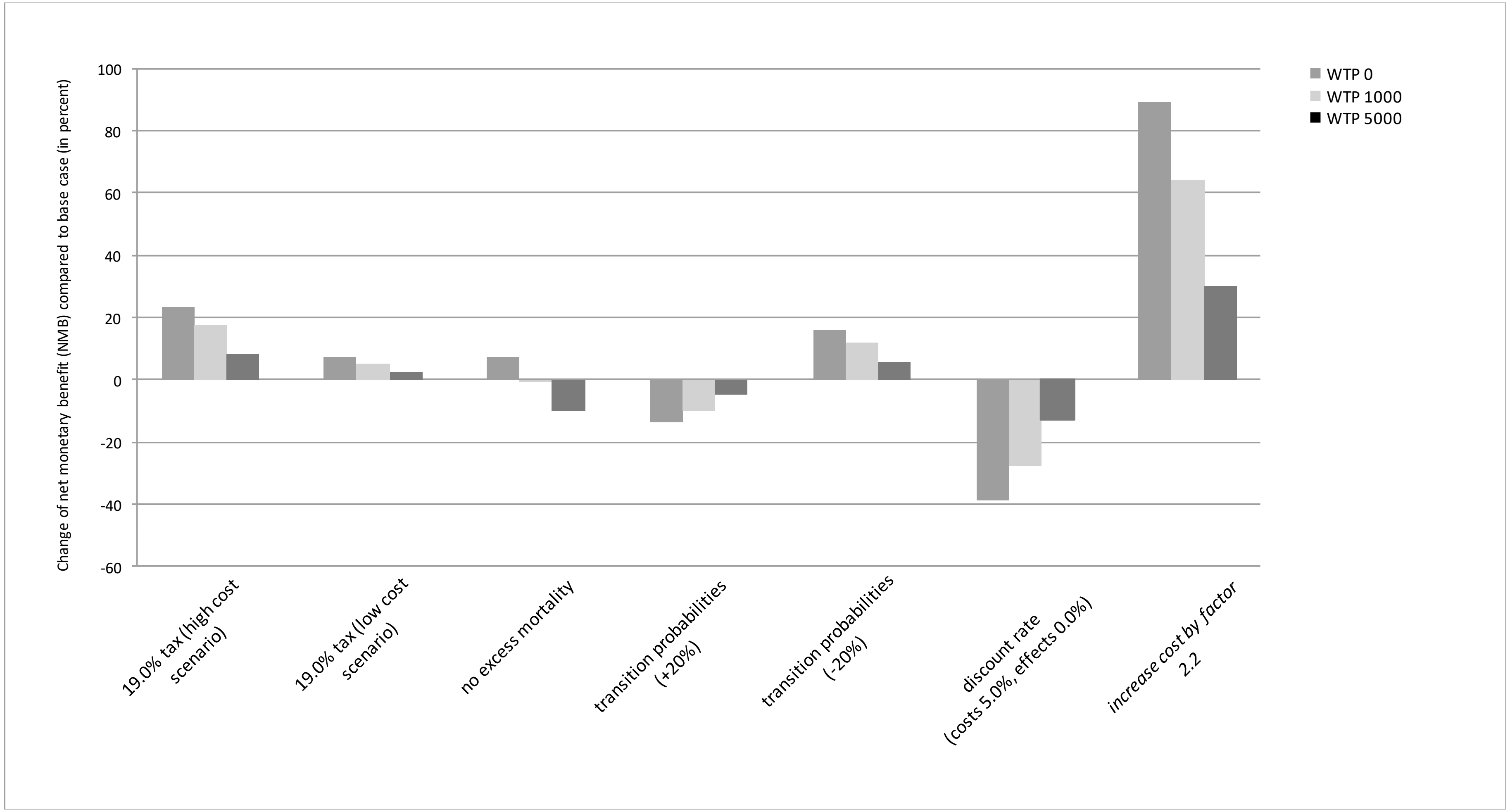

Univariate sensitivity analyses included evaluating the impact of (i) variability in parameter estimates of transition probabilities (varied by 20%), (ii) no excess mortality in adulthood for individuals who were obese during childhood, (iii) discount rates for cost data (0 and 5%), (iv) different excess cost estimates using a recently published bottom-up cost-of-illness study for Germany (Wolfenstetter et al. 2012), (v) taxing HP content formula by 19% according to cost of formula delivery (high- or low-cost scenario).
Base case WTP=0: €750.16, WTP=1,000: €1,047.72, WTP=5,000: €2,237.93 (NMB) (see Figure 4).
